# The ATTO 565 Dye and Its Applications in Microscopy

**DOI:** 10.3390/molecules29174243

**Published:** 2024-09-06

**Authors:** Yuheng Wu, René M. Williams

**Affiliations:** Molecular Photonics Group, Van ’t Hoff Institute for Molecular Sciences (HIMS), Universiteit van Amsterdam, Science Park 904, 1098 XH Amsterdam, The Netherlands; shenlanyuyinzhe@gmail.com

**Keywords:** high-resolution microscopy, fluorescent Rhodamine dye, stimulated emission depletion microscopy, fluorescent labeling, fluorescence correlation spectroscopy, 3D single-molecule tracking

## Abstract

ATTO 565, a Rhodamine-type dye, has garnered significant attention due to its remarkable optical properties, such as a high fluorescence quantum yield, and the fact that it is a relatively stable structure and has low biotoxicity. ATTO 565 has found extensive applications in combination with microscopy technology. In this review, the chemical and optical properties of ATTO 565 are introduced, along with the principles behind them. The functionality of ATTO 565 in confocal microscopy, stimulated emission depletion (STED) microscopy, single-molecule tracking (SMT) techniques, two-photon excitation–stimulated emission depletion microscopy (TPE-STED) and fluorescence correlation spectroscopy (FCS) is discussed. These studies demonstrate that ATTO 565 plays a crucial role in areas such as biological imaging and single-molecule localization, thus warranting further in-depth investigations. Finally, we present some prospects and concepts for the future applications of ATTO 565 in the fields of biocompatibility and metal ion detection. This review does not include theoretical calculations for the ATTO 565 molecule.

## 1. Introduction

**Figure 1:**
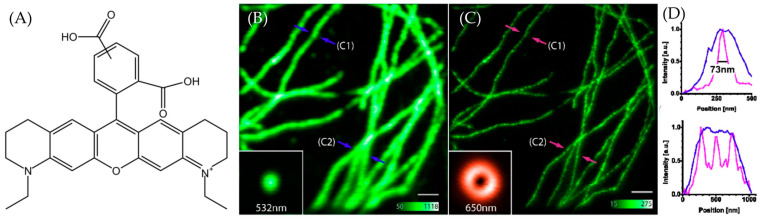
(**A**) The structure of ATTO 565. (**B**) The confocal image of PtK2 cells. C1 and C2 are the two selected representative locations. The fibers at C1 are relatively sparse, while at C2, they are denser, with three clusters stacked together. The intensity of green in the main image represents photon density, while the inset directly depicts the shape and color of the excitation light. The FWHM at (**B**) is shown in (**D**) with blue lines. (**C**) The STED image of PtK2 cells. C1 and C2 are the same position as in (**B**). The intensity of green in the main image represents photon density, while the inset directly depicts the shape and color of the STED depletion light. The full-width-half-maximum (FWHM) at (**C**) shown in (**D**) with pink lines. (**D**) The line graphs show the FWHM at the arrow location. The blue arrows (and lines) mark the confocal observations, while the pink/red arrows mark the STED observations. Upper image for C1; lower image for C2. It can be observed that the confocal microscope is unable to distinguish the number of fibers at location C2, whereas the STED microscope shows three peaks, corresponding to three fibers. Taken with permission from [5].

Fluorescent dyes are important tools across various domains such as biological imaging, cell tracking, and molecular probing. They play an essential role in advancing modern life sciences and materials science. In recent years, there has been a continuous surge in the research and development of novel fluorescent dyes [1]. As a type of Rhodamine dye, ATTO 565 shows notable traits, including intense absorption, high fluorescence quantum yield, and exceptional thermal and photo-stability [2]. These remarkable properties have rendered it extensively applicable in the field of single-molecule detection applications and high-resolution microscopy [3]. The structure of ATTO 565 is shown in Figure 1A. ATTO 565 is used in stimulated emission depletion (STED) microscopy. To overcome Abbe’s diffraction limit, stimulated emission is employed to deplete the fluorescent state. This generates focal regions of molecular excitation significantly smaller than the diffraction limit and significantly increases the resolution [4]. ATTO 565 is a suitable fluorescent dye in STED due to its superior performance. Wildanger et al. used ATTO 565 as a fluorescent labeling dye for immunofluorescence staining of mammalian PtK2 cells. Compared to the confocal image, the STED image clearly shows the structures and spaced fibers of cells [5], as shown in Figure 1B,C.

Besides its contribution to STED, ATTO 565 is also used in visually evaluating the drug delivery potential and studying the mechanism of drug delivery [6]. All these applications reveal that ATTO 565 is a promising fluorescent dye with great potential. In this review, the applications of ATTO 565 in various types of microscopy techniques are explored, revealing the significant capabilities of ATTO 565 in assisting researchers to explore the microscopic world.

## 2. The Properties of the ATTO 565 Dye

### 2.1. Chemical Properties

**Figure 2:**
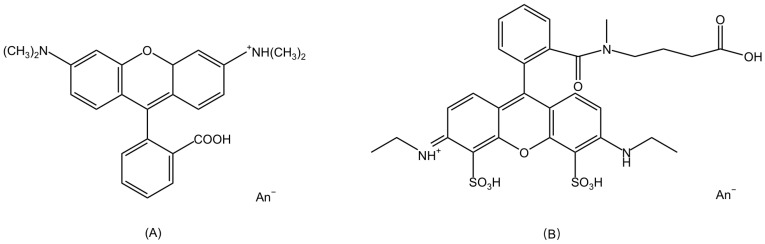
(**A**)The structure of Rhodamine B. An^−^ indicates anion. (**B**) The structure of ATTO 532.

Rhodamine dyes, as organic compounds, are a type of fluorescent dyes commonly used in biological, chemical, and fluorescence microscopy research. The molecular structures of Rhodamine dyes are based on a xanthene core which acts as the chromophore [7]. The primary differences between Rhodamine dyes lie in the various substituents on the xanthene framework. For example, there are two dimethylamino substituents on the xanthene core of Rhodamine B and two ethylamine groups for ATTO 532, as shown in Figure 2A,B.

There is a delocalized π system in the xanthene structure. When a molecule absorbs a photon, it undergoes a π → π* transition, subsequently emitting fluorescence during the de-excitation process [7]. Close to the xanthene ring, most Rhodamine dyes have a carboxyl substituent (or a similar substituent), which has a significant influence on the fluorescent properties of Rhodamine dyes.

**Figure 3:**
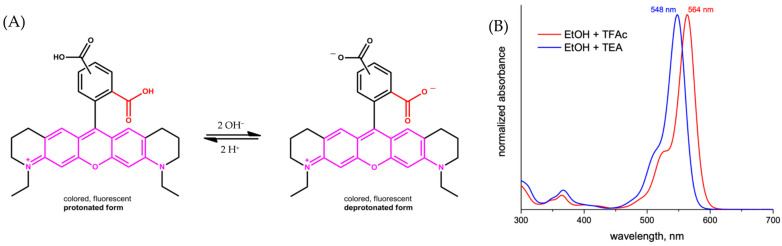
(**A**) The form changing of ATTO 565 in ethanol. (**B**) The absorption spectra of ATTO 565 in protonated and deprotonated form [8].

**Figure 4:**
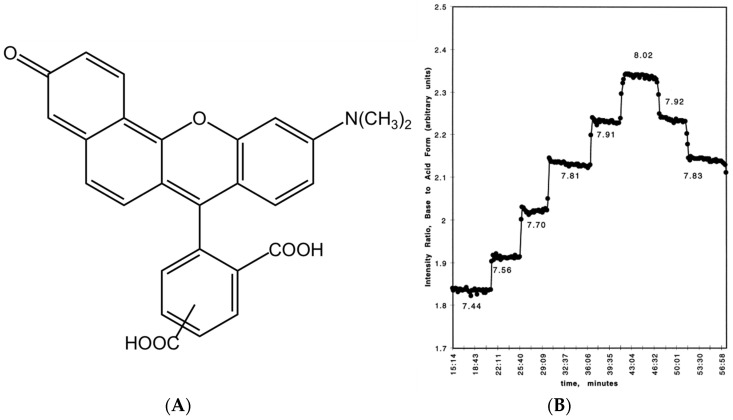
(**A**) The structure of SNARF-1C. (**B**) The intensity ratio of base to standard acid form. Taken with permission from [9].

For ATTO 565, the carboxyl group can dissociate to form a proton and the carboxylate under a basic protonic solvent environment, for example, when ATTO 565 is dissolved in ethanol (Figure 3A) [8]. The protonation of the carboxylate group leads to a shift in the absorption spectrum (Figure 3B) and reduces its emission intensity. This is the principle of the first kind of pH sensor. Grant et al. utilized the property of Rhodamine materials to develop a pH sensor centered around Seminaphthorhodamine-1 carboxylate (SNARF-1C) (Figure 4A) [9]. Under the same parameters and two known pH values, the ratio of the fluorescence intensities generated is always consistent. This allows for the measurement of all pH values relative to a standard acidic pH, with the fluorescence intensity ratio of the Rhodamine molecules used as a calibration. For example, Figure 4B shows the fluorescence intensity ratios of SNARF-1C measured in a phosphate-buffered saline (PBS) environment. Although there is a lack of relevant research, considering the structural similarity between ATTO 565 and SNARF-1C, and most importantly, because ATTO 565 also contains a benzoic acid group, ATTO 565 also has potential as a pH meter.

The second method for preparing Rhodamine dyes as pH meters utilizes the reversible ring-opening reaction of Rhodamine dyes. Li et al. synthesized a Lyso-DR molecule containing a Rhodamine structure [10]. This molecule features an intramolecular lactam structure that does not fluoresce in the closed form but emits fluorescence upon ring-opening in acidic environments. Although ATTO 565 does not have a lactam structure, it can still undergo a similar reversible ring-opening reaction at different pH values.

**Figure 5:**
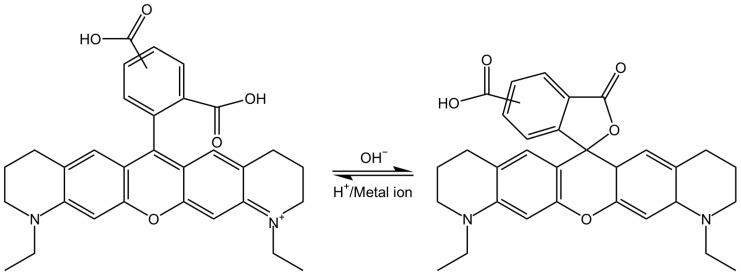
The structural switching of ATTO 565 in different conditions [8].

**Figure 6:**
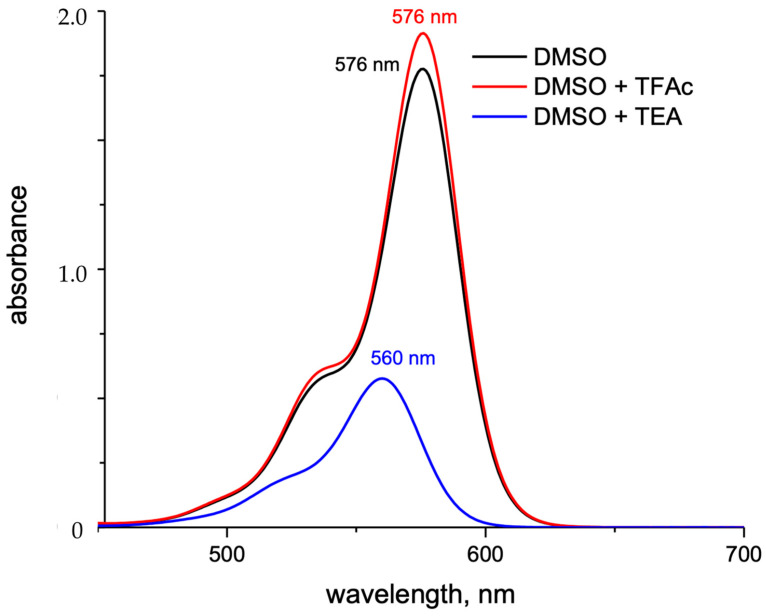
The UV–Vis absorption spectra of ATTO 565 in open ring form (DMSO + TFAc) or closed form (DMSO + TEA) [8].

In an alkaline environment, the carboxyl group loses a proton, leading to a nucleophilic attack on the central carbon atom to form a five-membered lactone structure. This closed-form structure interrupts the xanthene chromophore, generally causing Rhodamine dyes not to exhibit absorption or emission peaks within the visible light range. In comparison, in acidic or neutral environments, the xanthene structure remains in its original state, which is referred to as the open form. The open form of Rhodamine demonstrates superior fluorescence performance [11]. ATTO-TEC GmbH measured the absorption spectra of ATTO 565 in open form and closed form, shown in Figure 3B. They used trifluoroacetic acid (TFAc) and triethylamine (TEA) to influence the structure of ATTO 565. The switch of ATTO 565 between open and closed forms is shown in Figure 5 [8]. The result shows that the maximum absorbance of the open form is about four times higher than that of the closed form: the closed form of ATTO 565 barely absorbs and emits light (Figure 6). The structural conformational changes between the open and closed forms allow Rhodamine dyes to be used in different experimental conditions because their fluorescent properties can be controlled by altering pH or light exposure conditions, and this makes them highly valuable in fields such as cell biology, molecular biology, and biological imaging [12].

Lampidis et al. have already demonstrated that certain Rhodamine dyes exhibit selective accumulative biotoxicity to cancer cells without harming normal cells due to the higher plasma membrane potential of carcinoma cells. This implies that ATTO 565 also holds significant potential for in vivo experiments [13].

### 2.2. Photophysical Property

**Figure 7:**
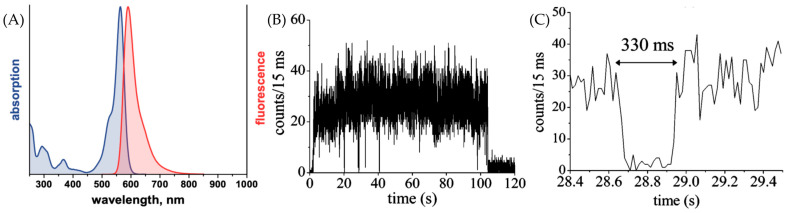
(**A**) The absorption and fluorescence spectrum of ATTO 565 [14]. (**B**) Fluorescence time trace of a single molecule of dye ATTO 565. Several dark states could be found before the molecule was photobleached. (**C**) Specific fluorescence intensity trace of (**B**) from 28.4 to 29.4 s which shows intermittency. Taken with permission from [15].

The fundamental characteristics of ATTO 565 were assessed by ATTO-TEC GmbH [14]. The emission and absorption spectra of ATTO 565 are shown in Figure 7A. The wavelength of the strongest absorption peak is ~564 nm, which is the origin of its name. The wavelength of the emission maximum is 590 nm. There is a clear Stokes shift for ATTO 565. This makes the use of ATTO 565 convenient for fluorescence excitation experiments as it reduces the influence of excitation light on the emission spectrum. The molar absorptivity of ATTO 565 is 1.2 × 10^5^ M^−1^ cm^−1^. This high absorptivity coefficient indicates that ATTO 565 possesses high sensitivity, enabling it to effectively absorb light even at low concentrations. The fluorescence quantum yield of ATTO 565 is 90%, which implies that ATTO 565 efficiently converts the absorbed energy into fluorescence emission, even under low concentration conditions of the analyte, producing a strong fluorescence signal. The optical data are shown in Table 1.

To gain insights into the properties of the dye, researchers employed single-molecule fluorescence spectroscopy to acquire typical emission intensity time traces of individual ATTO 565 molecules on a glass surface in a normal environment. The fluorescence time trace of dye 565 is shown in Figure 7B [15]. The intensity fluctuations are obvious. Several intermittencies happened before the final photobleaching at 105 s. During these intermittencies, the molecule stops emitting fluorescence, and this change in intensity is usually named “blinking”. When the trace is magnified at 28 s, one blinking event lasts 330 ms, as shown in Figure 7C [15].

**Figure 8:**
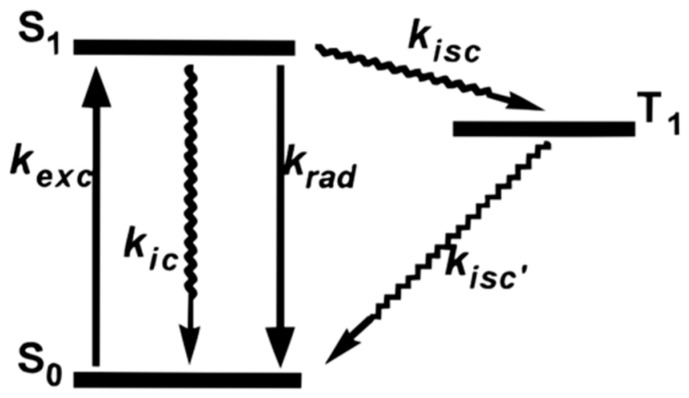
Three electronic states are proposed as one of the explanations for blinking. S_0_ is the ground state. S_1_ is the first excited state. Molecule could go through radiative relaxation and go back to S_0_ from S_1_ with the emission of fluorescence. Refer to the end of the review for meanings of other symbols. The molecule could also go through the intersystem crossing and go to the first triplet excited state T_1_ state then relax to S_0_ without any fluorescence which implies the dark state. k_exc_: Excitation rate constant, k_ic_: Internal conversion rate constant, k_rad_: Radiative decay rate constant, k_isc_: Intersystem crossing rate constant, k_isc_’: Reverse intersystem crossing rate constant. Straight arrows indicate that the process involves radiation (i.e., photons), while curved arrows represent non-radiative processes. Taken with permission from [16].

Some possible explanations for the blinking phenomenon have been proposed. One common explanation is the three electronic state theory shown in Figure 8. When the molecule absorbs the energy from the photon, it is excited from ground state S_0_ to the first excited state S_1_. After a very short excited state lifetime, it goes back to the S_0_ state with the emission of radiation. This is the reason for the “on time” of fluorescent dye molecules. However, there is a possibility that the molecule converts from the S_1_ to the triplet excited state T_1_. It will stay in T_1_ with a longer excited state lifetime than in S_1_ since conversion back to the ground state from T_1_ is spin forbidden. In the end, the molecule relaxes to S_0_. This pathway proceeds entirely through intersystem crossing and is completely non-fluorescent, manifesting as a molecular dark state on a macroscopic scale. Also, intensity fluctuations in some cases may result from environmental variations, such as minor temperature changes, which can impact the absorption spectrum [16].

**Figure 9:**
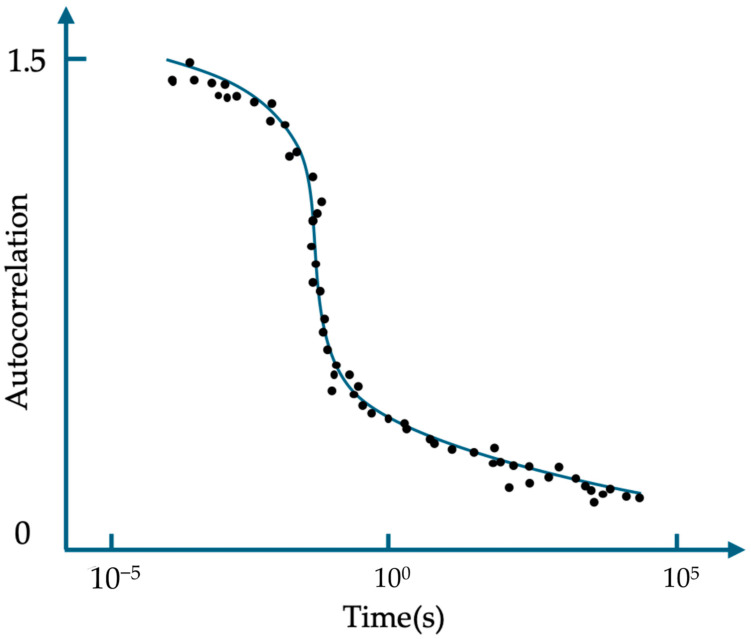
Simulation diagram of the autocorrelation function of the fluorescence signal of fluorescent molecules.

Further research, however, has revealed some evidence suggesting that the blinking phenomenon of ATTO 565 may not necessarily follow the three electronic state theory. Yeow et al. used the data of fluorescence intensity time trace to make the autocorrelation curve for ATTO 565 with an excitation wavelength of 543 nm [15]. Figure 9 is a schematic representation of the simulated autocorrelation function of fluorescent dye fluorescence, which closely resembles the actual experimental results of ATTO 565. Fitting the curve using Formula (1) allows us to obtain some key parameters. Using the formula brought forward by Krichevsky et al. to fit the curve [17], the average triplet state lifetime yielded a value of 6 μs. A more specific explanation of the autocorrelation curve is in Section 3.4. In Formula (1), Neff denotes the average number of fluorescent molecules in ROI, F is the fraction of molecules that are in the triplet state, and w is a parameter about the volume of the ROI.

G(t) = {[1 − F + Fexp (−t/τ_tri_)]/[(1 − F) N_eff_]} × [(1 + t/τ_diff_) (1 + t/w^2^ τ_diff_)^0.5^]^−1^ (1)

However, the measured dark state time of a single molecule in Figure 7C is 3 × 10^5^ μs, and it is tens of thousands of times bigger than τ_tri_. This indicates that the three electron states may not explain the blinking of ATTO 565 well.

**Figure 10:**
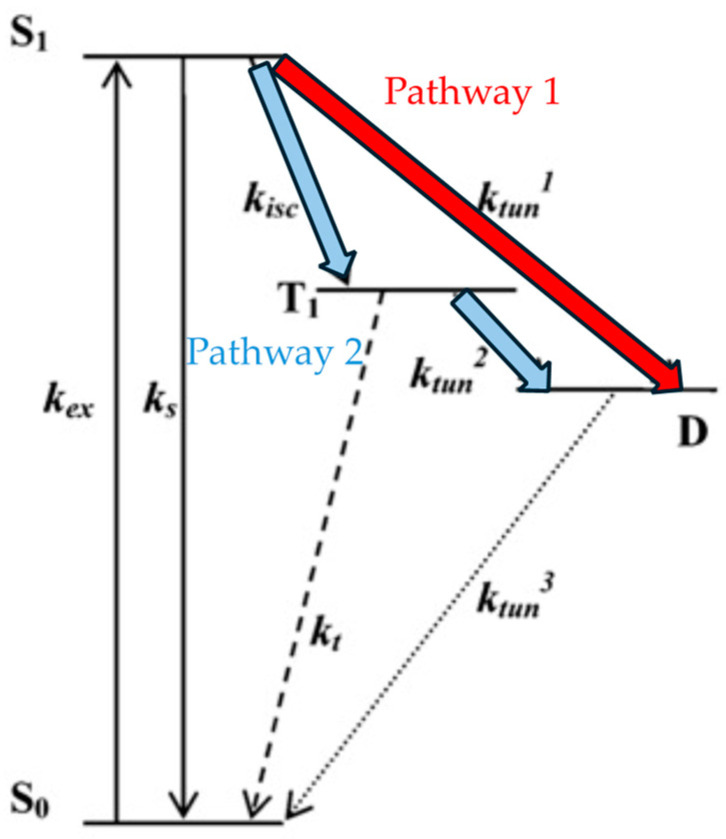
Four electronic states explain the blinking phenomenon. Refer to the end of the document for meanings of other symbols. k_ex_: Excitation rate constant, k_isc_: intersystem crossing rate constant, k_s_: Intersystem crossing rate constant from the singlet state, $k_{tun}^{1–3}$: Tunneling rate constant, k_t_: Triplet quenching, S_0_: Ground singlet state, S_1_: First excited singlet state, T_1_: First excited triplet state, D: Dark state. Reprinted (adapted) with permission from [15].

Another proposed theory is based on four electronic states shown in Figure 10. After being excited to S_1_, the molecule has two pathways to reach the dark state D. One involves intersystem crossing to T_1_ followed by electron tunneling to the dark state, while the other pathway directly involves electron tunneling to the dark state. Yeow et al. conducted Monte Carlo simulations to test the validity of the dual-pathway model and found that reproducing the observed fluorescence intermittency behavior in the experiments requires the simultaneous consideration of both pathway 1 and pathway 2 [15].

**Figure 11:**
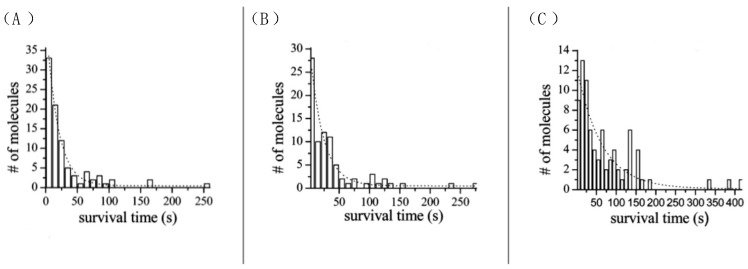
ATTO 565 molecules were put into lighting at different intensities. (**A**) 1136 W/cm^2^, (**B**) 568 W/cm^2^, (**A**) 284 W/cm^2^, the average bleaching times are 18.2 s (**A**), 21.8 s (**B**), and 63.0 s (**C**), # represents number. Reprinted (adapted) with permission from [15].

As a fluorescent dye, ATTO 565 will suffer photobleaching under high-intensity lighting. ATTO 565 molecules were placed in different-intensity lighting conditions and we tested their bleaching time histogram, as shown in Figure 11 [15].

**Figure 12:**
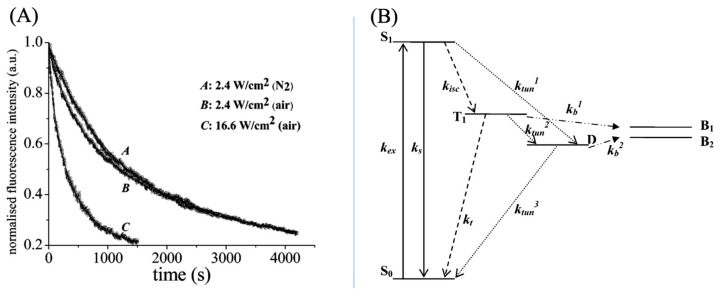
(**A**) Temporal intensity profile of ATTO 565 photobleaching. A and B exhibit a trend of monoexponentially decay and C is biexponential decay. k_1_ = 5.1 × 10^−4^ s^−1^ and k_2_ = 2.9 × 10^−3^ s^−1^ for A. k_3_ = 2.2 × 10^−3^ s^−1^ and k_4_ = 1.2 × 10^−2^ s^−1^ for B. k_5_ = 8.5 × 10^−4^ s^−1^ for C. (**B**) Energy-level diagram used to explain photobleaching. k_ex_: Excitation rate constant, k_isc_: intersystem crossing rate constant, k_s_: Intersystem crossing rate constant from the singlet state, $k_{tun}^{1–3}$: Tunneling rate constant, k_t_: Triplet quenching, k_b_: bleach rate constant, S_0_: Ground singlet state, S_1_: First excited singlet state, T_1_: First excited triplet state, D is the radical state and B is the bleached state. Refer to the end of the document for meanings of other symbols. Reprinted (adapted) with permission from [15].

To further elucidate the mechanism of photo-bleaching in ATTO 565, researchers monitored changes in fluorescence intensity over time in different media. The groups with air condition exhibit biexponential decay and the group with nitrogen condition exhibits monoexponentially decay, as shown in Figure 12A. A four-electron energy level hypothesis has been proposed to explain biexponential decay, as shown in Figure 12B. There are two different pathways of photobleaching. In the first one, the molecule will form a radical D state, and the bleaching rate is determined from the rate of molecule transfer from T_1_ to B_1_ which is the bleached state, and D to B_2_. In the other pathway, the molecule would not form a D state, and the bleaching rate is determined only by k_b_. From the macroscopic perspective, the decay rate of ATTO 565 molecular populations in air is determined by two rate constants due to the two pathways.

The bleaching mechanism in the air is related to oxygen. “The quenching mechanism is most likely due to an oxygen-dependent reaction whereby reactive singlet oxygen (^1^O_2_) formed from the reaction between T_1_ and triplet oxygen (^3^O_2_) can attack and eventually destroy the molecules” (see p. 1732), as stated by Yeow et al. [15]. When there is a lack of oxygen in the environment, bleaching may occur due to reactions with the surrounding matrix in T_1_/D [15].

## 3. The Application of ATTO 565 in Microscopy

### 3.1. Applications of ATTO 565 in Confocal Microscopy

#### 3.1.1. Utilizing ATTO 565 for Assessing the Effectiveness of Nanostructures

ATTO 565 can be directly employed in fluorescent imaging of nanometer-sized structures. The concept of “Lab-on-a-chip” represents a groundbreaking approach that consolidates various chemical and biological analysis functions onto a single compact chip, designed for handling extremely minute liquid volumes, down to less than a pico-liter. This chip comprises numerous microchannels, necessitating a convenient method for assessing the effectiveness of these microchannels [18]. Due to its excellent fluorescence performance, ATTO 565 stands out in fulfilling this requirement.

**Figure 13:**
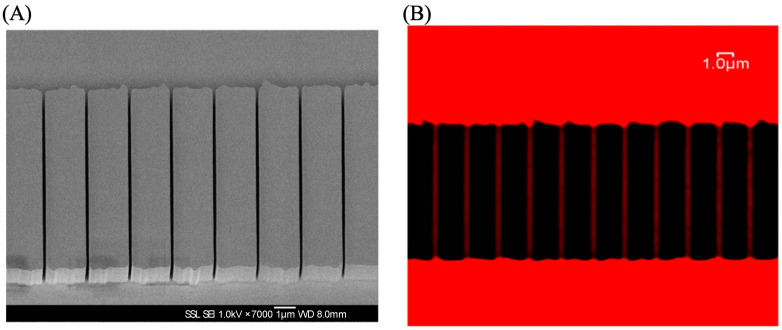
(**A**) The SEM image of the nanochannel. (**B**) The confocal microscope image of the nanochannel. The fluorescence of ATTO 565 in red demonstrates the connectivity of microchannels. The diameter of the pipeline is 100 nanometers. Reprinted (adapted) with permission from [19].

Wang et al. utilized ATTO 565 in conjunction with a confocal microscope to assess the performance of Proton Beam Writing (PBW), a technique for fabricating and etching micro and nanostructures [19]. Poly(methyl methacrylate) (PMMA) was selected as the material of the whole nanofluidic structure because it exhibits good reproducibility in PBW [20], and its transparency facilitates fluorescence detection. They initiated the process by employing a 2 MeV photon beam to create nanofluidic channels with a width of 100 nm and a depth of 2 μm, followed by the design of inlet and outlet channels. Subsequently, a 1 nM ATTO 565 solution was introduced into the nanosystem using a syringe pump. Laser lines, generated at 543 nm with a HeNe laser, were then employed to excite ATTO 565. Fluorescence correlation spectroscopy (FCS) was utilized to determine the time it takes for the liquid to first flow out of the nanoscale pipeline and to completely flow out, aiding in guiding the practical applications of nanoscale pipelines. Additionally, confocal microscopy captured side-view images of the nanoscale pipelines. The result is presented in Figure 13, where it can be observed that the diameter of the nanoscale pipelines is approximately 100 nanometers, and its shape is perfectly straight.

#### 3.1.2. Utilizing ATTO 565 for Assessing the Effectiveness of Biostructures

**Figure 14:**
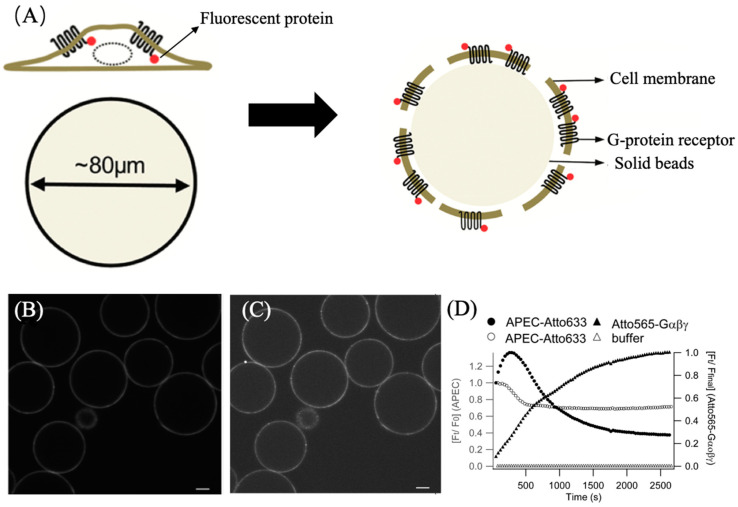
(**A**) Cover the cell membrane inside-out on agarose beads using stir and wash. WGA on the beads immobilizes the cell membrane. (**B**) Confocal microscope image of Gαβγ-ATTO 565 on the beads. (**C**) Confocal microscope image of APEC-ATTO 633 on the beads. Scale bars of (**B**,**C**) are 20 μm (**D**) Shows the time-responsive fluorescence curve. Black circle curve illustrates the binding level of APEC to GPCR over time. The hollow circle curve illustrates the dynamic binding of APEC and GPCR in the presence of buffer. The black triangle curve shows the binding of Gαβγ and GPCR indicated by ATTO565 fluorescence. Reprinted (adapted) with permission from [21].

ATTO 565 is commonly utilized in biology to label and visualize biological molecules, cell structures, and biological processes for research and monitoring purposes. For example, Roizard et al. employed ATTO 565 to visualize the binding of G-protein receptors (GPCRs) to G proteins on the kidney cell membrane and determined their reaction kinetics parameters [21]. GPCRs are a class of protein receptors widely present on the cell membrane that can interact with G proteins [22]. To introduce fluorescent dyes like ATTO 565 into the membrane and measure the reactions using microscopes effectively, it is necessary to transform the cell membrane into solid-supported membranes, which involves fixing biological membranes on a solid surface. Researchers achieved this by immobilizing cells on agarose beads coated with wheat germ agglutinin (WGA) and then flipping the cell membrane inside-out onto the agarose beads through stirring, as depicted in Figure 14A. WGA is a type of protein that can bind with glycans on the plasma membrane and subsequently fix the cell membrane, facilitating ligand binding. Within the membrane, A_2A_R fused with mCitrine (A_2A_R-Citrine) is located. A_2A_R represents a type of GPCR, and citrine is employed as a marker. These components are generated by a plasmid within the cell.

The A_2A_-AR agonist (APEC) is combined with ATTO 633 [23]. ATTO 633 is another Rhodamine dye like ATTO 565 but with different absorption and emission wavelengths. The G protein Gαβγ is combined with tris-NTA-Pro8-ATTO 565, a fluorescent probe described in detail in Section 3.2.3. Three fluorescent dyes were employed in this experiment to clearly distinguish GPCR, ligand, and G protein. Firstly, the membrane was exposed to a 60 nM APEC-ATTO 633 solution, followed by exposure to 17 nM Gαβγ-ATTO 565, and fluorescent data-recording commenced. The excitation wavelengths for ATTO 633 and ATTO 565 are 633 nm and 561 nm, respectively. Confocal microscope images are presented in Figure 14B,C, confirming successful binding of the ligand and G protein to the cell membrane. Figure 14D illustrates the binding status of the fluorescent dyes with GPCRs. The fluorescence intensity data revealed a reverse effect of G proteins on the binding equilibrium between the ligand and GPCRs. With the addition of G protein-ATTO 565, the fluorescence intensity of ATTO 633 on the cell membrane gradually increased to its maximum, indicating peak binding of APEC and GPCRs. Subsequently, the increased fluorescence intensity of ATTO 565 suggests that as more G proteins bind to GPCRs, APEC gradually dissociates from the ligand, with a significantly higher degree of dissociation compared to when G proteins are absent. This implies that the presence of G proteins increases the dissociation constant of the ligand–receptor complex. Therefore, the dissociation constant (KD) of the ligand–GPCR complex could be calculated using the Cheng–Prusoff equation.

K_i_ = IC_50_/(1 + L/KD) (2)

Ki is the dissociation constant of the inhibitor with receptor (ATTO 565-Gαβγ-GPCR) and L is the concentration of APEC-ATTO 633. The KD is calculated as 12 ± 3 nM and this implies that the G protein indeed exerts an inhibitory effect on APEC-GPCR.

The experiments described above show the dynamic bonding events between G proteins or selective ligand such as APEC and biological receptor GPCRs, which can be monitored at the ~100 nm scale using fluorescent dyes at nanomolar concentration.

#### 3.1.3. Application of ATTO 565 in Multilevel Imaging in 3D Structure

**Figure 15:**
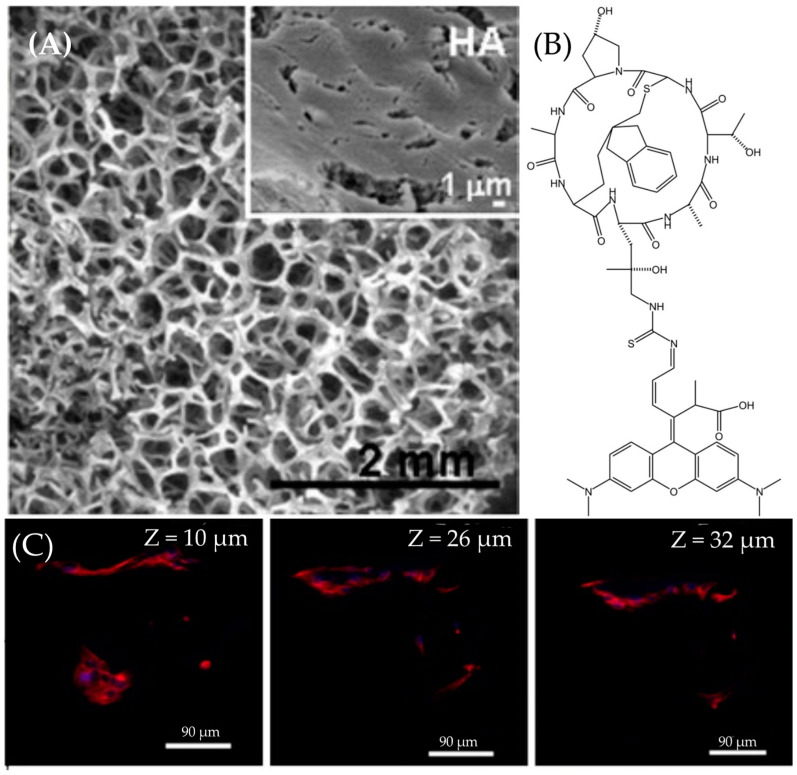
(**A**) The SEM image of HA pores. The white part is the HA backbone. The diameters of pores are ranged from 1 μm to 400 μm. The subfigure shows the tiniest pores with about 1 μm diameter. (**B**) The chemical structure of Rhodamine–phalloidin. These two molecules are conjugated by a thiourea. (**C**) These three images demonstrate the capability of ATTO565 to localize HOS cells at different depths in space from 10 μm to 32 μm. The red part is the fluorescence emitted by ATTO565, indicating the location of HOS cells. Reprinted (adapted) with permission from [25].

ATTO 565 is utilized in conjunction with microscopy for imaging nanoscale 3D structures. In tissue engineering, Poly-ε-caprolactone (PCL) materials have proven particularly beneficial in promoting the healing and recovery of tissues, such as bones, owing to their internal micron-sized bubbles that provide space for cell adhesion and growth [24]. Cicuéndez et al. effectively demonstrated the growth status and distribution of HOS cells on the hydroxyapatite (HA) using ATTO 565 [25]. HA was synthesized through the reaction between calcium nitrate tetrahydrate and triethylphosphite. To render HA porous, Pluronic F127 surfactant was added as a macro-pore former during the sol-gel synthesis process. The SEM image of HA, shown in Figure 15A, depicts a structure replete with porous features ranging in size from 1 to 0.4 mm. Researchers seeded the HOS cells in the pores at a density of 2 × 10^5^ cells mL^−1^ and incubated them for 4 days. Subsequently, the cells were washed with PBS and fixed in paraformaldehyde [25].

Filamentous Actin (F-actin) is a versatile globular protein found abundantly in most eukaryotic cells, serving as a vital component of the cellular cytoskeleton [26]. Essentially, F-actin constitutes the cytoskeleton; so, its localization using fluorescent dyes enables the localization of the cell itself. Fluorescence immunostaining is the method employed to label F-actin. Phalloidin serves as the mediator to mark F-actin with ATTO 565. Initially extracted from poisonous mushrooms, phalloidin has been shown to bind to amino acid residues 117, 119, and 355 of F-actin, demonstrating a strong affinity for F-actin [27]. Furthermore, through organic chemistry methods, Rhodamine dyes can be conjugated to phalloidin via a thiourea linkage. The chemical structure of Rhodamine–phalloidin is depicted in Figure 15B. Additionally, Rhodamine–phalloidin probes are readily available for purchase. Cells on HA were incubated with ATTO 565-phalloidin and subsequently washed with PBS. The excitation wavelength was set at 563 nm. Confocal microscopy images are presented in Figure 15C. By adjusting the depth of the focal plane, images of various depths of the sample could be captured, demonstrating the replication of cells on HA and the formation of interconnected cell groups at varying depths within the porous structure [25].

**Figure 16:**
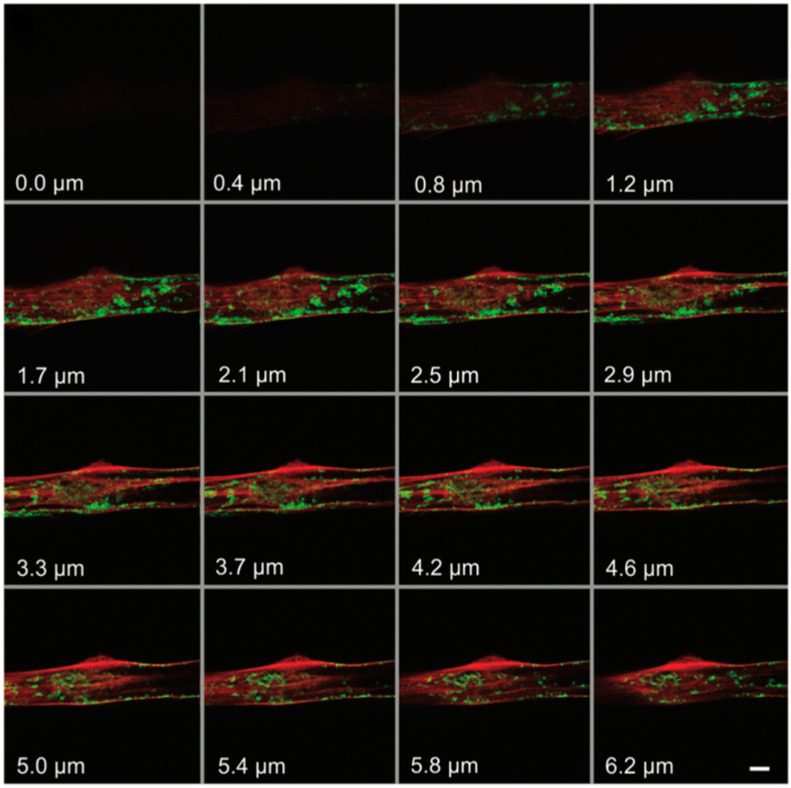
The confocal microscope images of F-actin. The red emission light comes from ATTO 565-phalloidin-F-actin and green emission light comes from GFP-dysferlin-caveolin. The number marked in the lower left corner is the Z value, which represents the depth of the focal plane. Reprinted with permission from [29].

F-actin has been shown to be associated with the repair of plasma membranes [28]. Marg et al. also used ATTO 565-phalloidin to demonstrate this phenomenon. The researchers employed a confocal microscope to irradiate a 2.5 × 2.5 μm area of the plasma membrane of primary human myoblasts at maximum power (10 mW diode laser, 488 nm laser line) for 38 s, inducing damage to the cell membrane. Subsequently, the cells were fixed with formaldehyde–PBS solution and blocked with BSA. Following this, F-actin staining was performed using phalloidin–ATTO 565. To capture different levels of the cell, a z-scan was conducted during the experiment. The results, depicted in Figure 16, revealed F-actin accumulation at the wound site, forming a “dome” structure [29]. This evidence supports the notion that F-actin plays a crucial role in plasma membrane repair. Conversely, findings from the localization of green fluorescent protein (GFP)-dysferlin indicate that Caveolin does not accumulate at the injury site, suggesting that Caveolin is not involved in cellular repair.

### 3.2. Applications of ATTO 565 in STED

#### 3.2.1. Applications of ATTO 565 in CW STED

Ernst Abbe, the renowned German physicist, proposed the formula for the limitation of optical microscope resolution, expressed as d = λ/(2nsinθ), where d represents the resolution, λ is the wavelength of light, and n × sinθ is the numerical aperture (NA). This equation delineates the limit for the resolution of optical microscopy, suggesting that optical microscopy cannot distinguish two points separated by less than half the wavelength of light. It is widely accepted that the best resolution achievable by optical microscopy is approximately 200 nm [30]. However, the advent of stimulated emission depletion (STED) microscopy, introduced by Stefan W. Hell, surpasses this limit and sets a new standard for microscope resolution.

The fundamental principle of STED involves the addition of high-power laser light to a conventional laser scanning microscope, inducing molecules to undergo stimulated emission, returning from the excited state (S_1_) to the ground state (S_0_) without fluorescence radiation [30]. By restricting the region emitting fluorescence, the resolution is significantly enhanced.

Rhodamine dyes, including ATTO 565, are widely employed in STED microscopy because of their exceptional fluorescence characteristics. Firstly, the power of the STED beam is 10,000 to 100,000 times greater than the power of the excitation beam used in a confocal microscope, which can lead to a strong photobleaching effect [31]. In this case, ATTO 565 is suitable for STED measurements due to its high photostability. After 30 min of exposure to 2.4 W/cm^2^ beam, 55% of ATTO 565 molecules were photobleached. For comparison, 60% to 70% of cyanine dye are photobleached in 30 min under a 14 mW/cm^2^ beam [32]. Secondly, according to the principle of STED explained above, a suitable fluorescent dye for STED should have a strong depletion ability, which means that it should easily stop emitting light under the STED depletion beam. The rate of the depletion can be described as σ_S10∗_(λ) = λ^4^E(λ)φf/(8πcn^2^τ), where λ is the wavelength of STED light and E is the emission spectrum of dyes at λ. σ is the cross section of the depletion, which is positively correlated with the depletion rate [33]. The other parameters are not related to this topic. From this equation, it can be intuitively observed that, in order to maximize σ as much as possible, the optimal value of λ will appear slightly after the emission peak, influenced by the parameters λ to the fourth power and E(λ). However, selecting STED λ too close to the emission peak introduces a new issue: increased noise. In most cases when using a microscope, it is advisable to avoid situations where the wavelength of the light source is similar to that of the light emitted by the fluorescent dye; that is also the reason a Stokes shift is preferred. In summary, a suitable dye for STED needs to have a long and strong red tail to allow users to more comfortably choose the STED wavelength, avoiding noise and improving depletion efficiency. ATTO 565 has an evident red tail in fluorescent spectra, which is another reason to be used in STED.

ATTO 565 contributes to the research in the biochemistry domain, which always needs high-resolution images at the organelle scale. Here, a typical example is presented. The classical fluid mosaic model indicated that proteins are embedded in the lipid bilayer and can move or remain fixed. However, it could not explain why many membrane proteins with similar structures cluster together such as receptors and syntaxins [34]. In this case, Willig et al. used STED microscopy with ATTO 565 to study the fine structure of syntaxin clusters on the cell membrane. They first used ultrasound treatment to remove the upper part of a PC12 cell and left a cell sheet. The cell sheet was then fixed in 4% paraformaldehyde in phosphate-buffered saline (PBS) to prevent crosslinking of syntaxins [35]. Then, the cell sheet was incubated with HPC-1 which acted as primary antibodies to combine syntaxin 1A/B [36]. Then, they chose ATTO 565-coupled goat-anti-mouse lgG as secondary antibodies to combine with HPC-1.

**Figure 17:**
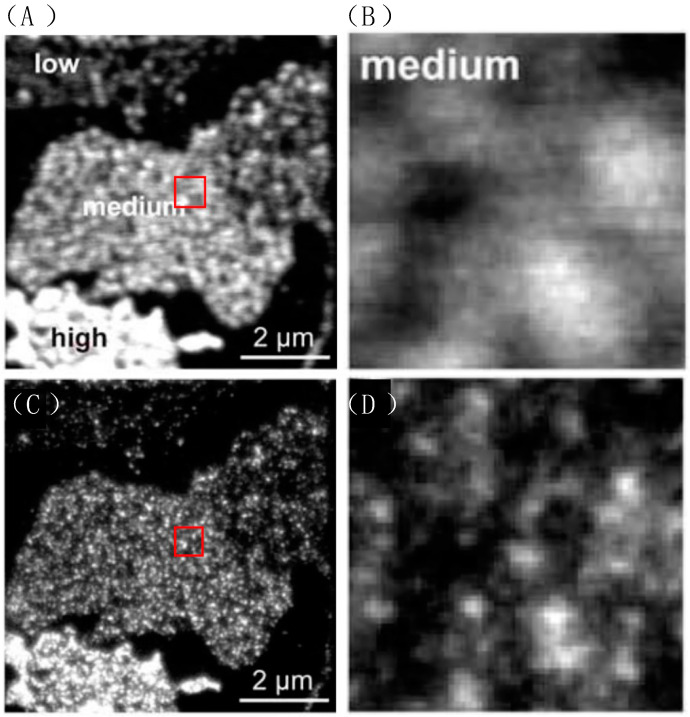
The confocal and STED microscope image of syntaxin-GFP. Low, medium and high indicate the fluorescent protein expression level. (**A**,**B**) The confocal images. Membrane sheets showing highly variable syntaxin levels were distinguishable by their staining intensities and were occasionally found within the same field of view. (**C**,**D**) In STED mode, photos taken at the same location which is marked with the red box in (**A**,**C**), show significantly improved resolution. Reprinted with permission from [38].

To avoid the excitement by STED light, usually control *λ*_STED_ < *λ*_EXC_. In this case, a common 532 nm laser diode was chosen as the exciting light, and a 647 nm line of a krypton laser was chosen as CW STED light. The clusters labeled with fluorescence were displayed as light spots in the microscopic images. A similar research conducted by Sieber et al. marking clusters with green fluorescent protein (GFP) also showed the high resolution of STED. The result is shown in Figure 17 and it is clear that STED microscopy employing fluorescent dye achieves a level of resolution significantly surpassing that of conventional optical light microscopy. The points of clusters are separated much better in STED. With the same method, Sieber et al. calculated that the average of diameter of the cluster is 55 nm, with each cluster containing approximately 90 syntaxins [37].

#### 3.2.2. Applications of ATTO 565 in T-Rex STED

**Figure 18:**
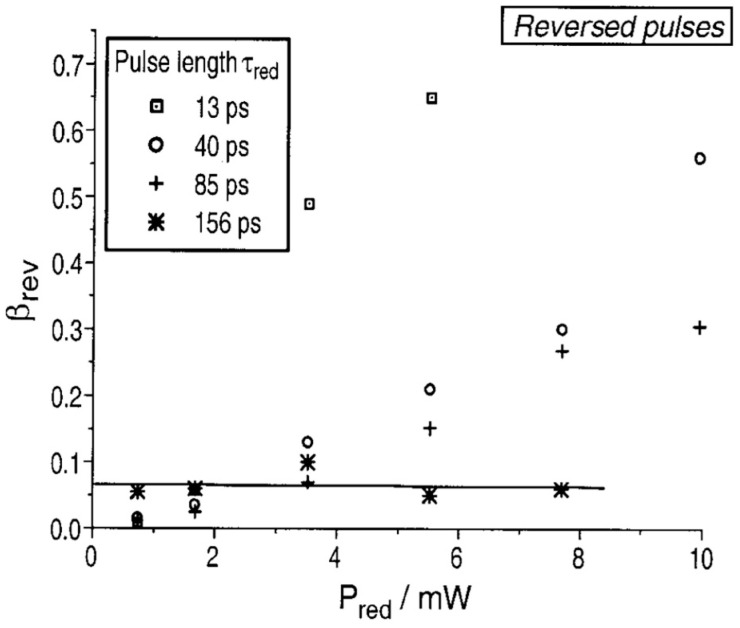
Variation in bleaching efficiency β with STED light intensity at different pulse durations. In the same time intervals, the extent of dye bleaching increases with the increasing intensity of STED red light. Reprinted with permission from [40].

The resolution of STED microscopy has been well defined by the following equation:

Δr = λ/[2NA (1 + I_STED_/I_S_)^0.5^] (3)

where Δr represents the FWHM, indicating the resolution, and Is is the intensity of STED light at which half of the molecules are quenched. NA stands for numerical aperture and λ is the wavelength. The resolution is primarily determined by I_STED_, which is the maximum intensity of the STED light [39]. From Equation (3), it is clear that increasing the intensity of STED light is a method to enhance the resolution. However, Dyba and Hell conducted a photobleaching experiment of RH-414 under STED conditions and obtained a general result for fluorescent dyes, indicating that increasing STED light intensity also leads to the photo-bleaching of fluorescent dyes, as shown in Figure 18.

In this scenario, the use of ATTO 565 dye combined with T-Rex STED addresses the issue of photobleaching. The key characteristic of T-Rex is the pulsed nature of both the excitation light and STED light. In this setup, molecules in the triplet state have more time to return to the ground state during the gap between pulses, rather than immediately absorbing more photons and undergoing photobleaching. The typical lifetime of T_1_ is 1 μs, and the traditional STED pulse repetition rate is 80 MHz, with a gap time of 0.0125 μs. Donnert et al. reduced the pulse repetition rate to 0.25 MHz, resulting in a gap time four times longer than τ_T_. This extended gap time provides molecules with more opportunities to relax to the ground state, effectively preventing further photobleaching [41].

**Figure 19:**
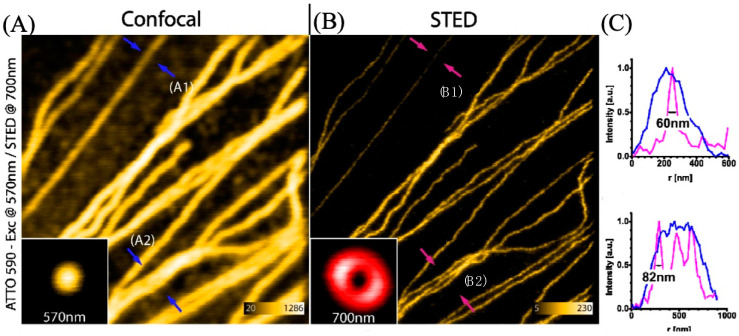
(**A**) The image of the tubular fibers was obtained with a confocal microscope. A1 and A2 are two selected typical positions, marked by blue arrows. At A1, the fiber distribution is sparse with only one fiber, whereas at A2, the fiber distribution is dense with three fibers. (**B**) The image of the same tubular fibers was scanned with a STED microscope. B1 and B2 correspond to the same positions as A1 and A2, marked by pink/red arrows. (**C**) The line graphs show the FWHM at the arrow location. Upper image for A1 (blue line) and B1 (pink line) and lower image for A2 (blue) and B2 (pink). It is evident that the red STED spectrum distinguishes the three fibers, while the confocal spectrum fails to do so. Scale bar: 1 μm. Reprinted/adapted with permission from [5].

Wildanger et al. used ATTO 565 with a T-Rex STED microscope to observe the tubular network of mammalian PtK2 cells [5]. Using anti-mouse IgG and sheep anti-mouse IgG, the method to connect ATTO 565 with the target tubular network is the same as in the case in Section 3.2. The excitation light and T-Rex STED light were both generated by a supercontinuum source, with a pulse length of 82 pm generated by a master oscillator. An interference filter was employed to selectively filter the excitation light wavelength to around 532 nm. T-Rex STED light is effective when the wavelength is set around the red tail of the dye’s emission spectrum, within a range of 20 nm. In this case, the T-Rex STED light needs a prism-based wavelength selector, which is more precise. The STED wavelength is selected as 650 nm. The result is shown in Figure 19. The images obtained with STED are significantly clearer than those from the confocal microscope. In the confocal microscope, the FWHM at arrow A1 is approximately 240 nm, whereas the FWHM value for T-Rex STED is 60 nm. This means that the resolution of T-Rex STED is four times that of the confocal microscope [5]. Although the T-Rex STED reduced the likelihood of photobleaching at 565 nm, the supercontinuum source in T-Rex STED spectroscopy cost about EUR 400,000. Considering such a high price, exploring alternative substitutes for the instrument can be considered.

#### 3.2.3. ATTO 565 in TPE-STED Microscopy

**Figure 20:**
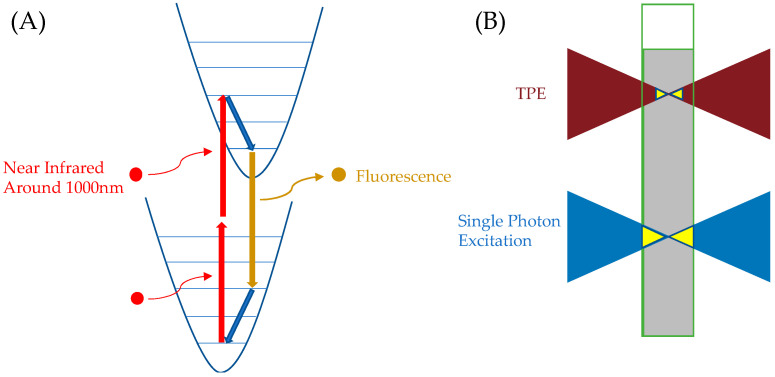
(**A**) TPE excitation process of fluorescent molecules. Red curved arrows represent the excitation process of photons interacting with the molecule, red straight arrows indicate the process of the molecule transitioning to a higher energy level, blue arrows represent vibrational relaxation, and yellow arrows represent the fluorescence process. (**B**) Compared to conventional excitation light, TPE excites a smaller volume of the region.

The excitation pathway of ATTO 565 mentioned above primarily involves a single-molecule process. That is, a high-energy photon interacts with the ATTO 565 molecule, promoting it to a higher energy level. In contrast, emerging two-photon excitation fluorescence microscopy involves the interaction of two photons with ATTO 565. As shown in Figure 20A, two near infrared photons with a lower energy compared to a normal excitation photon (which could be 400–700 nm) interact with the fluorescent molecule in 10^−16^ to 10^−18^ s and excite the molecules. After that, the molecular process is almost identical to the single-photon excitation process. TPE has two advantages. First, the near-infrared light used in TPE penetrates biological samples more easily. Second, TPE excitation requires a high-density photon region. As shown in Figure 20B, regular excitation light can excite the sample throughout the entire conical area, causing ATTO 565 molecules to fluoresce, while TPE excites the sample only near the focal point. This makes TPE suitable for 3D sample microscopy [42].

Moneron et al. used a 1060 nm pulsed TPE light source with 7–15 mW power as the central excitation light for the STED microscope and a 200 mW continuous STED ring-shaped light as the depletion beam to illuminate the nucleus of mammalian PtK2 cells. In the cell nucleus, the transcription regulator NFκB was labeled with ATTO 565 via primary and secondary antibodies. It is worth noting that to increase the probability of TPE, the photon density of the TPE light must be higher than that of regular excitation light, which can lead to significant photobleaching effects on ATTO 565. Therefore, a pulsed excitation light was used. More details can be obtained from the original paper, but we can conclude that the resolution of TPE microscopic images was significantly improved with the aid of STED [43].

#### 3.2.4. Optimized Fluorophores with ATTO 565

**Figure 21:**
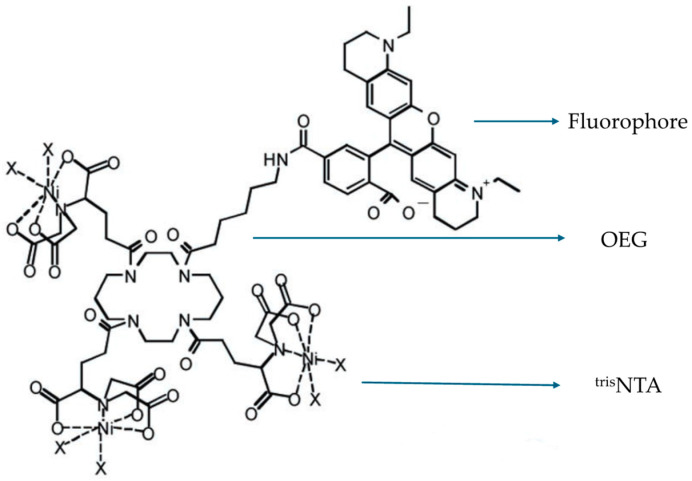
The structure of ^tris^NTA-OEG-ATTO 565. The fluorophore could be any kind of Rhodamine dye. Reprinted (adapted) with permission from [44].

In the cases mentioned above, the fluorescent dye ATTO 565 is conjugated to the target by immunoassay. The drawback of this method is that the combination of dye and target is irreversible and inflexible, especially when the dye is photobleached. The optimization of fluorophores is necessary. Lata et al. pioneered a noncovalent fluorescent labeling method [44]. The designed fluorescent molecule is tris-nitrilotriacetic acid (^tris^NTA)-oligo ethylene glycol (OEG)-fluorophores as shown in Figure 21. The fluorophores applied were Rhodamine dyes and are connected to OEG using organic chemistry. The OEG serves as the connector and imparts different properties to the molecule depending on its length [45]. On the other end, ^tris^NTA acts as a chelator. The carboxyl groups chelate with metal ions such as Ni and Cu. Based on the properties of these metal ions, they also possess two electronic orbitals that can chelate with the imidazole structure on the His-tag added to the protein [46]. This forms the binding chain from the fluorescent dye ATTO 565 to the target molecule.

**Figure 22:**
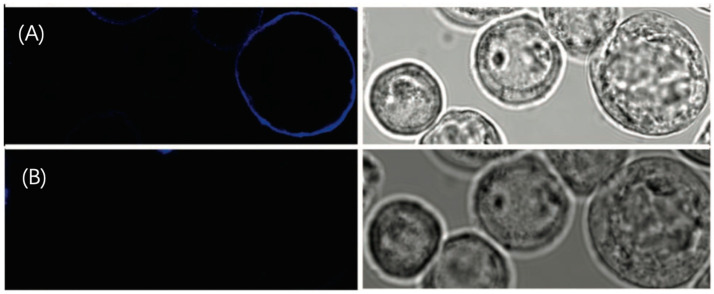
(**A**) Image of Sf9 cells stained by FEW646tris-NTA. (**B**) The fluorescent dye is washed away after incubation in 100 mM imidazole, indicating the reversible stain. On the left side are the confocal fluorescent images, and on the right side are the transmission images of Sf9 cells. Reprinted (adapted) with permission from [44].

There are two advantages of this noncovalent strategy. First, the reaction between NTA and the target protein is reversible. If an imidazole solution is added to the environment, the ^tris^NTA bound to the protein can be detached [47]. Researchers stained the Sf9 cells with FEW646-^tris^NTA, and the fluorescent dye was washed away after adding a 150 mM imidazole solution. The result of imidazole substitution is shown in Figure 22, which reveals the reversibility of this fluorescence dye staining process. This provides a new solution for reducing photobleaching effects: by replacing with imidazole, the already photobleached dye can be washed off and then replaced with a new dye.

**Figure 23:**
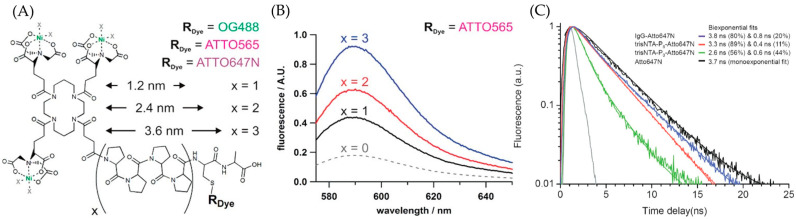
(**A**) The structure of ^tris^NTA-PPII-ATTO 565. X indicates the monomer number of PP-II. (**B**) The fluorescence spectrum shows that fluorescence intensity is positively correlated with the connector length. (**C**) The fluorescence spectrum indicates that when the monomer number reaches eight, the fluorescence intensity is essentially equivalent to that of the free fluorophore. Reprinted (adapted) with permission from [45].

The other advantage of the NTA-based fluorophore comes from the adjustable-length connector. Studies have shown that transition metal ions could quench the fluorophore [44]. This phenomenon is due to the electron transfer from metal ions to the fluorophore. The process could be indicated as F* + M^II^ → F^−^ + M^III^, where F means fluorophore and M is a transition metal such as Cu or Ni. The thermodynamic data ${\Delta G}_{eT}^{o}$ support the feasibility of this reaction, while the deprotonation of nitrogen stabilizes the uncommon trivalent metal ions [48]. However, studies have also shown that the length of the connector directly affects this process. Grunwald et al. designed an NTA-based ATTO 565 fluorophore that uses rigid (polyproline-II) PP-II helices as the connector, as shown in Figure 23A [45]. At different connector lengths, the fluorophore exhibits varying emission spectra. Experimental results indicate that as the length of PP-II increases, the fluorescence intensity becomes stronger, demonstrating a lower probability of quenching by transition metal ions. The result also shows that when the monomer number reaches eight, the effect of the connector in reducing fluorescence quenching reaches saturation (Figure 23B,C). Thus, selecting eight PP units as connectors would facilitate the attainment of the highest possible fluorescence brightness yield for ATTO 565.

In summary, the ^tris^NTA-connector-ATTO 565 fluorophore can be reversibly stained onto the target, exhibiting a replaceable effect, and the length of the connector is adjustable, enhancing its fluorescence quantum yield. High-resolution imaging, such as tracking individual receptors in a native cell, can also be achieved [49].

#### 3.2.5. Usage of ATTO 565 with Other Dyes in Dual-Color STED Microscopy

Kempf et al. used ATTO 565 and Dyomics 485 to label transport proteins and synaptic proteins in rat brain tissue and visualized them using dual-color STED microscopy to determine the distribution of these distinct proteins. Dual-color STED microscopy uses multiple excitation light sources to simultaneously excite two fluorescent molecules, and employs different wavelengths of depletion light to selectively suppress the fluorescence emissions of these two molecules [50].

**Figure 24:**
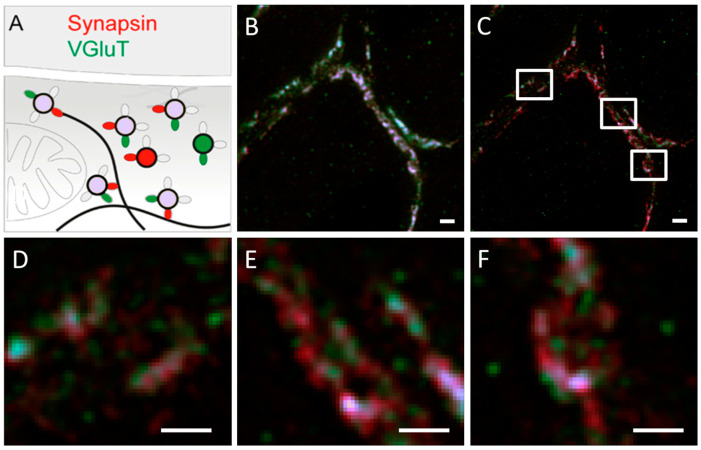
(**A**) The luminescence colors of the protein under different binding conditions. The green oval represents ATTO 565, the green circle represents VGluT, the red oval represents Dyomics 485, and the red circle represents synapsin, while the product of the reaction between VGluT and synapsin is represented by a purple circle. (**B**) Confocal microscope image. (**C**) STED microscope image, showing more details. (**D**–**F**) Shows an enlarged view of the area highlighted by the white square. Scale bar 500 nm with a pixel size of 19 nm. Reprinted with permission from [50].

The VGluT1 labeled with ATTO 565 appears green, while the synapsin labeled with Dyomics 485 appears red. When VGluT1 and synapsin are co-labeled and interact, the combined fluorescence appears purple, as shown in Figure 24A. The confocal and STED microscopy images are shown in Figure 24B and C, respectively. It is evident that relying solely on confocal microscopy results could lead to incorrect conclusions, whereas the STED microscopy images reveal additional information on isolated synapsin.

### 3.3. ATTO 565 in Single-Molecule Tracking (SMT)

Much information is averaged out when observing many molecules simultaneously. Instead of observing a group of molecules at the same time, an SMT technique only records the information of individual molecules [51]. With SMT, one can obtain more information from the molecular perspective, such as the position, diffusion coefficient, and photobleaching condition [52].

#### 3.3.1. The Basic Principle of 2D Single-Molecule Tracking

**Figure 25:**
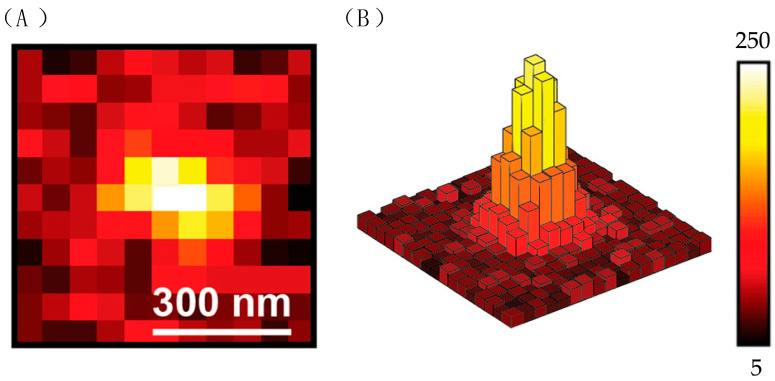
(**A**) A scheme of an individual fluorescent signal emitted by a single molecule. The intensity in the middle is the highest, which indicates the location of the molecule. (**B**) A scheme shows the distribution of intensity in one signal. Reprinted (adapted) with permission from [53].

To make the target molecule visible, a fluorescent dye is always conjugated with the target. The dye is excited and emits fluorescence, which indicates the location of the target molecule. In an SMT experiment, a traditional microscope such as a confocal microscope or widefield fluorescent microscope is sufficient to record the fluorescence. As mentioned before, the diffraction limit makes the resolution limit of a traditional microscope around 200 nm. This means that in the image generated by CCD, the shape of one molecule is always a blurry circle with a diameter of at least hundreds of nanometers, as shown in Figure 25A. This is why in an SMT experiment, a very low sample density is required to minimize the overlap of two molecules.

The location of the molecule is reasonably regarded as the centroid of the signal, and the signal is divided into many pixels, as shown in Figure 25B. Light intensity is weighted along the *x*-axis using Function (4).

(4) $$\mu_{x}=[\sum_{i=1}^{M_{y}}\;\sum_{i=1}^{M_{x}}\;x_{i}\;I_{ij}]/[\sum_{i=1}^{M_{y}}\;\sum_{i=1}^{M_{x}}\;I_{ij}]$$

I_ij_ indicates the intensity of light on pixel (i, j). X and y are the coordinates of pixels along the *x*-axis and *y*-axis. μ_x_ is the coordinate where the signal is maximum. The calculation for μ_y_ is the same. The coordinate of the molecule is then (μ_x_, μ_y_) [52]. Then, the coordinates x and y are inputted into Function (5), which is a Gaussian distribution function:

(5) $$I(x,y)=4ln2N\exp\{-4ln2[({\left(x-\mu_{2}^{x}\right)}^{2}/w^{2}+{\left(y-\mu_{2}^{y}\right)}^{2}/w^{2})]\}$$

where N is the photon number hit at the point (x, y), and w is related to NA. After fitting the data into the function, the μx, μy can be obtained [52]. The track of a molecule can then be calculated after obtaining its position.

#### 3.3.2. The Application of ATTO 565 in 2D Single-Molecule Tracking

**Figure 26:**
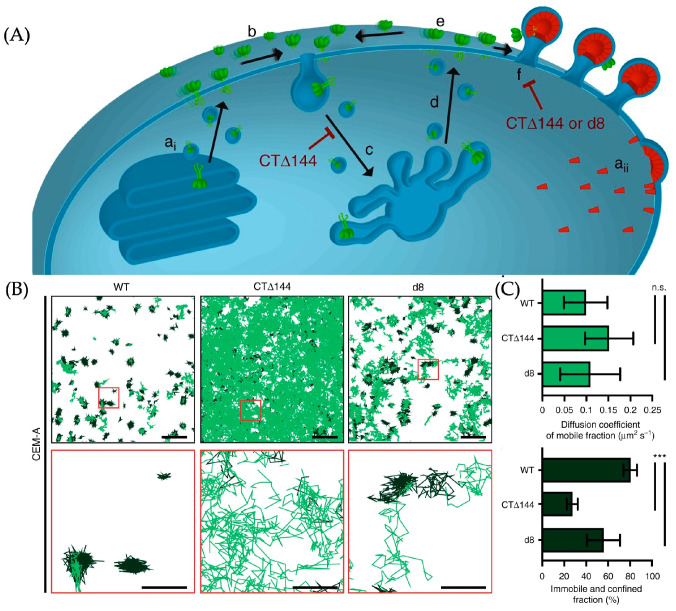
(**A**) The scheme of transportation and assembling of Env in the target cell. The green object is Env and it is finally confined at the Gap. a_i_ represents the synthesis of Env in the endoplasmic reticulum, a_ii_ represents the synthesis of Gag at the cell membrane, b indicates the diffusion of assembled Env trimers on the cell membrane, c and d represent the occasional recycling process of Env trimers, e represents the occasional endocytosis of trimers, and f represents the binding of Env trimers with Gag. (**B**,**C**) The result of the SMT. The displacement of WT-Env is smaller than others, indicating that the assembling of CTΔ144-Env-ATTO 565 and d8-Env-ATTO 565 on the Gap is obstructed. In (**B**), the first row of images shows an overhead view of multiple molecular trajectories, with representative regions highlighted by red boxes. The enlarged view of these regions is shown in the second row of images. n.s. indicates not significant using one-way ANOVA with Tukey’s post-test and *** indicates *p*  <  0.0001. Reprinted (adapted) with permission from [55].

The pathways of HIV assembly and replication in cells have attracted the attention of researchers. One essential step is the synthesis and transportation of envelope glycoprotein (Env), which is located on the outer surface of the HIV particle, to interact with the host cell membrane, allowing HIV entry into the host cell. It is generally accepted that Env is synthesized in the endoplasmic reticulum and transported to the cell membrane by vesicles, and then assembled on the polypeptide Gag, as shown in Figure 26A [54]. The principle of this process is still unclear, and it is believed that the long cytoplasmic tail of gp41 (Env-CT), which is a part of Env, is related to the assembling process.

Carmen et al. used ATTO 565 to mark the wild-type Env (WT-Env) and removed CT Env (CTΔ144-Env) on the cell and used SMT to record the track of the molecules, concluding that the Env-CT does affect the assemble of Env [55].

The researchers infected CEM-A cells with three groups of HIV. The first group consisted of the original HIV, inducing host cells to synthesize WT-Env. The second group was genetically edited HIV, causing host cells to synthesize CTΔ144-Env. The third group was also genetically edited HIV, leading to host cells synthesizing d8-Env.

The marking of the Env is also through immunolabeling. Anti-Env antibodies b12 are conjugated with ATTO 565 and incubated with the infected cells. Then, the diffusion track of marked Env is recorded by interferometric photo-activated localization microscopy (iPALM). The result is shown in Figure 26B. The molecules with a high estimated slope of the moment scaling spectrums are classified as mobile molecules and the track is shown in light green. The rest is classified as confined molecules and shown in dark green.

The track of CTΔ144-Env-ATTO 565 and d8-Env-ATTO 565 was shorter than that of WT-Env-ATTO 565, with a smaller diffusion coefficient. Conversely, WT-Env-ATTO 565 molecules were more trapped, i.e., more likely to combine with the Gap. This leads to the conclusion that Env-CT promotes the assembling of Env on the Gap.

#### 3.3.3. The Principle of Locating Molecules on *Z*-Axis

Traditional microscopes could only capture a two-dimensional image of the sample. The method of capturing multiple 2D images to depict 3D structures is still unsatisfactory. By introducing a cylindrical lens (CL) into the microscope system, the trajectory of molecules on the *Z*-axis can be recorded.

**Figure 27:**
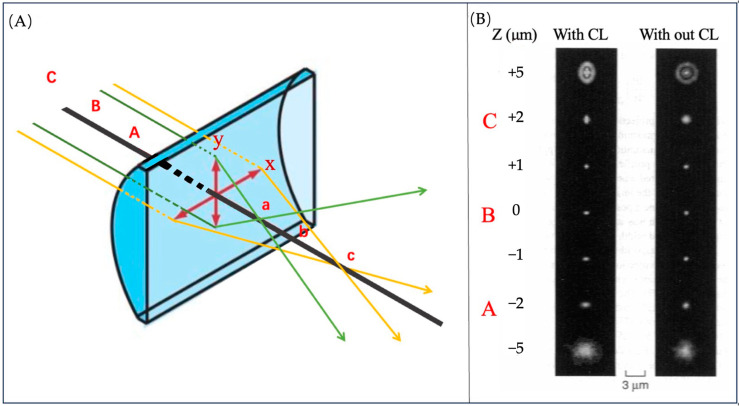
(**A**) CL has focus a for the *Y*-axis and focus c for the *X*-axis, and b is the middle point of them. On the other side, it has focus planes A, C and B. The red arrows indicate the x, y coordinate axes of the section. The green and yellow arrows represent incident light from different planes. (**B**) The image of the red fluorescent latex bead with and without a CL. Reprinted (adapted) with permission from [57].

Different from traditional convex lenses, the focal lengths of CL on the *X* and *Y*-axis are slightly different due to the different curvature [56], as shown in Figure 27A. Because of the different curvature, the shape of the object’s image corresponds one-to-one with its distance from the lens, as shown in Figure 27B. When the ellipticity of image is measured, the *Z* value can also be obtained.

#### 3.3.4. The Application of ATTO 565 in 3D Single-Molecule Tracking with Light Sheet Microscopy

Traditional microscopes have the light source and the eyepiece positioned on opposite sides of the object. Light travels through the object and enters the eyepiece, producing an image for the observer [58]. However, this optical setup has its limitations. Firstly, the resulting image often suffers from significant noise due to background light flooding into the eyepiece indiscriminately, resulting in a poor signal-to-noise ratio (S/N). Secondly, traditional light microscopes are constrained by Rayleigh and Abbe’s criteria, meaning they cannot resolve two points less than 200 nm apart [59].

**Figure 28:**
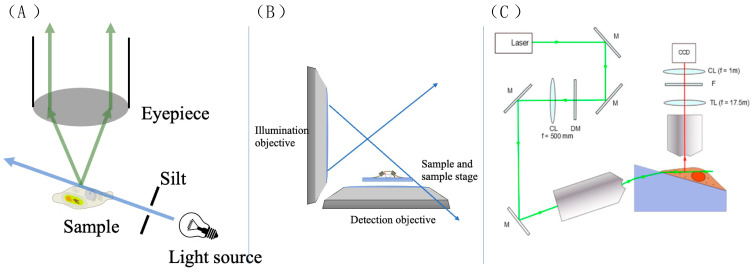
(**A**) The scheme shows the biggest difference between the LSM and traditional micro-scope. The light passes through the sample and does not directly go into the eyepiece. (**B**) Diagram of space constraint. (**C**) The LSM system used by Li et al. Reprinted (adapted) with permission from [61].

A reconfiguration of the optical components gives rise to the light sheet microscope (LSM), addressing the aforementioned drawbacks. Illustrated in Figure 28A, the illumination light is redirected away from the eyepiece, with only scattered or fluorescent light being observed. This creates a dark environment for the observer, minimizing noise. The illumination light passes through a slit, allowing only a thin sheet of light to illuminate the sample. This selective illumination reduces noise significantly, resulting in higher contrast compared to traditional microscopes. However, practical applications may impose spatial constraints on component arrangement [60]. For example, while arranging lenses vertically is common in LSMs, focusing light onto the sample can sometimes be challenging, as depicted in Figure 28B.

Li et al. assembled the LSM system shown in Figure 28C to observe epidermal growth factor (EGF) molecules on A549 cell membranes with ATTO 565 [61]. A cylindrical lens is put between the tube lens and the image sensor (CCD) to create two focal planes. The cell was placed on a Pellin–Broca prism to reduce space constraints for the lenses and make it easier to generate the calibration curve. The sample was tilted at 17.5° due to the prism.

To generate the calibration curve, the researchers added a drop of diluted 40 nm fluorescent beads solution (10 pM) on an A549 cell. Some of the beads were fixed on the cell membrane after 15 to 20 min of incubation. The observation system was moved horizontally while recording the images. The horizontal movement resulted in a change in the Z value of the observed beads. The relationship is shown in Function (6).

dz = sin(17.5π/180) dx(6)

**Figure 29:**
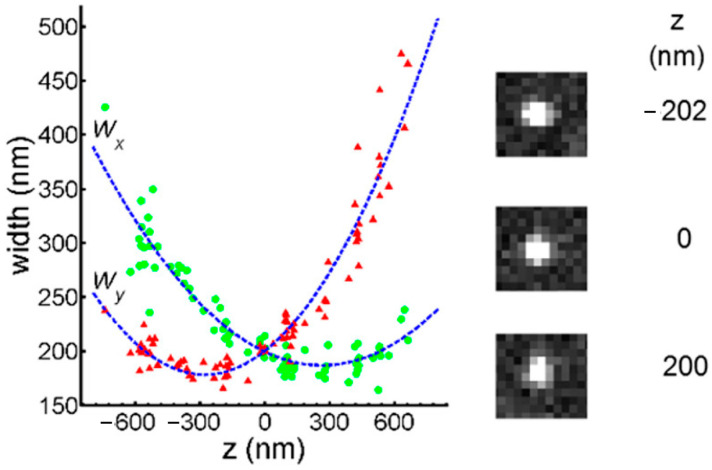
The calibration curve was measured by fluorescent beads and LSM. The image exhibits varying degrees of ellipticity at different distances from the focal plane. Each green dot represents the semi-major axis value of the Gaussian ellipse at the corresponding height (z), while the red triangles represent the semi-minor axis value. Reprinted (adapted) with permission from [61].

The scatter of PSF to Z and the calibration curve are shown in Figure 29.

The detection of EGF relies on the biotin/streptavidin system, with EGF-biotin and streptavidin-ATTO 565 readily available on the market. Each streptavidin molecule possesses four biotin binding sites [62]. Leveraging this robust biotin–streptavidin interaction, up to four ATTO 565 molecules can bind to a single EGF molecule. This approach not only enhances the fluorescent intensity of the target molecule compared to other labeling methods discussed in this review but also extends the duration of observation.

**Figure 30:**
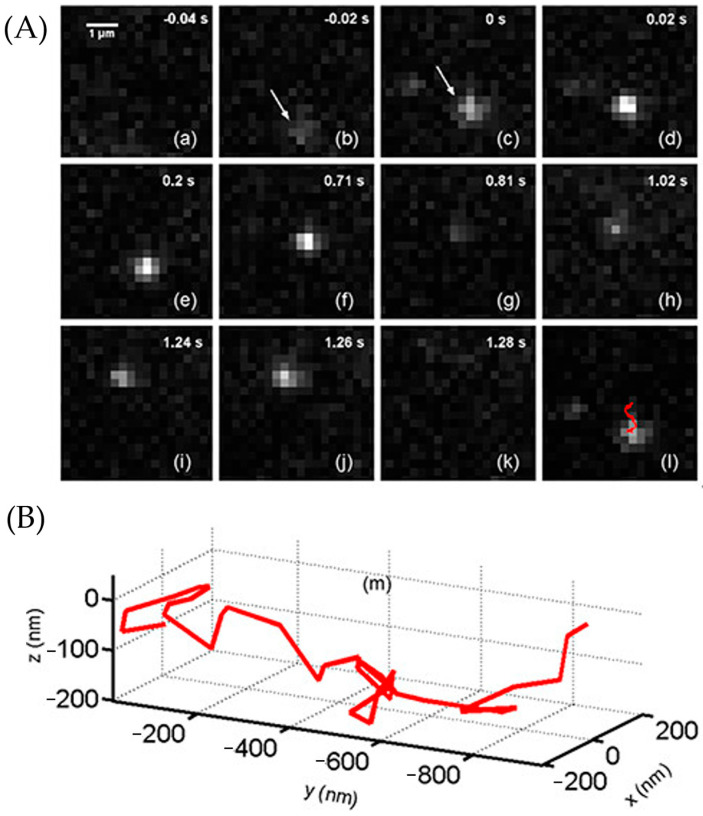
(**A**) The 2D image time sequence (**a**)–(**k**) was shot by the LSM. The molecule went into the ROI at −0.02 s (**b**) and out of the ROI (or was photobleached) at 1.28 s (**k**). Panel (**l**) marked the particle’s trajectory from 0 s to 0.8 s with a red arrow. (**B**) The 3D visualized trajectory of the molecule from 0 s to 8 s. After 8 s, the molecule was too deviated to simulate the trail. Reprinted (adapted) with permission from [61].

A549 cells were incubated with a solution containing 1.59 μM EGF-biotin-streptavidin-ATTO 565. Subsequently, the movement of individual EGF molecules was monitored using LSM. The image of EGF and its movement scheme are depicted in Figure 30A. At −0.02 s, a blurry molecule appeared within the field of view, indicating it was out of the focal plane. The movement trajectory from 0 s to 8 s is visualized in three dimensions in Figure 30B. After 1.28 s, the molecule either moved away from the region of interest (ROI) or underwent photobleaching.

### 3.4. ATTO 565 in Fluorescence Correlation Spectroscopy (FCS)

FCS is a technique that fully utilizes the fluctuation in equilibrium. Many observations in a chemical system start with an imbalance and end at the equilibrium state. However, FCS starts at the equilibrium. FCS records all fluctuations after the system reaches equilibrium and uses correlations to obtain information such as the viscosity, triplet state lifetimes, number of fluorophores, and so on [63].

**Figure 31:**
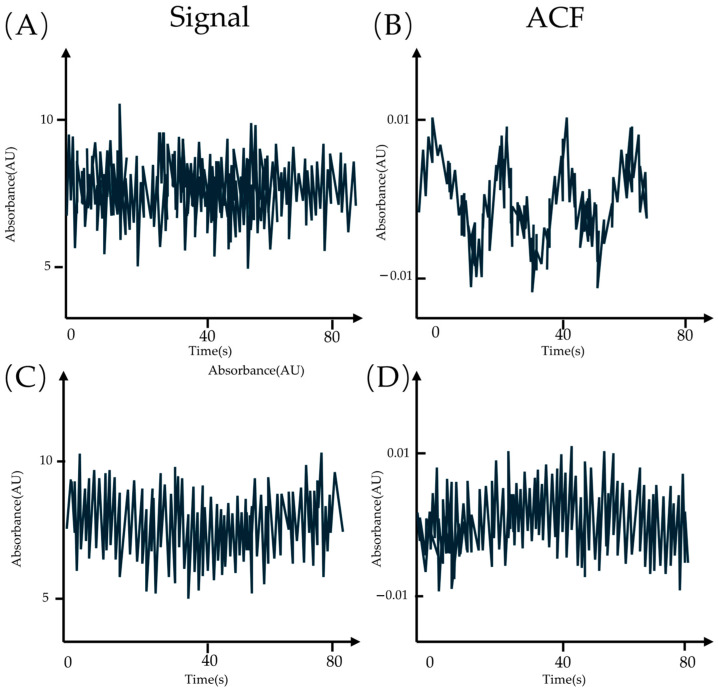
(**A**,**C**) The intensity–time line graph of the periodical signal and the noise. (**B**,**D**) The ACF of the signal and the noise.

For example, a series of periodical signals is shown in Figure 31A, and a series of noises in Figure 31C. It is hard to distinguish them from the intensity–time line graph. However, if transformed into the autocorrelation function form (ACF), it is straightforwardly observed that there is lot of information contained in the ACF of the periodical signal (Figure 31B,D).

If two variables, a and b, both vary with one parameter u, they are not correlated when they satisfy the following conditions:

<a(u) × b(u)> = <a(u)> <b(u)>(7)

<x> indicates the expectation of x. If these two variables are correlated to some extent, then Function (7) is not valid and a new variable could be defined:

G = <a(u)b(u)>/<a(u)> <b(u)>(8)

where G is the correlation coefficient, which measures the degree of correlation between parameters a and b. When G deviates from 1, parameters a and b are positively or negatively correlated. In an FCS experiment, parameters a and b could be the intensity of fluorescence and u the time. By reformulating Function (8) and fitting it with data, the desired parameters could be obtained. An example is introduced below.

Pan et al. used FCS to calculate the diffusion time of ATTO 565 in a PAAc solution [64]. The researchers dissolved ATTO 565 into PAAc solvent and used 543 nm laser lines to excite the fluorescent dye. There is only one group of parameters at a certain time; Function (8) is transformed into an ACF form:

G(t) = <a(u) a(u + t)>/<a(u)> <a(u + t)>(9)

Function (9) points out the correlation between parameter a and parameter a after time t. In this experiment, there is already a specific form of Function (10), which is written as follows:

G(t) = [Ae^−t/B^/(1 + A) + 1)](1 + t/td)^−1^(1 + t/K^2^td)^−0.5^/N + 1 (10)

where N is the number of fluorescent dyes in the ROI and td is the diffusion time of ATTO 565. A is the fraction of ATTO 565 which is in the triplet state, i.e., the dark state. B is the time of triplet state. After fitting the data into Function (10), the diffusion time can be obtained. The result is shown in Table 2.

### 3.5. Other Applications of ATTO 565

#### 3.5.1. Detection of Intracellular Structures by ATTO 565

By integrating Structured Illumination Microscopy (SIM) and the construction of fluorescent probes mentioned above, Han et al. developed an ATTO 565-based fluorescent probe for observing intracellular lysosomes with STED microscopy [65].

**Figure 32:**
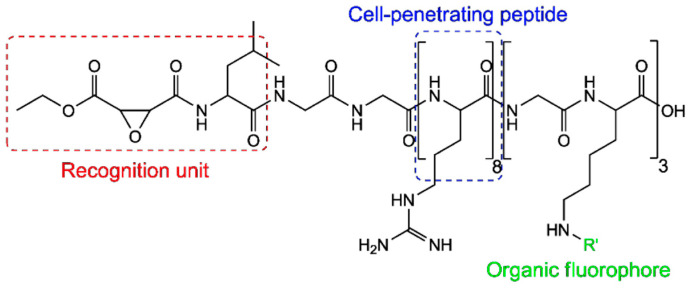
Structure of the ATTO 565 molecular probe for detecting intracellular structures. Re-used with permission from [65].

The structure of the fluorescent probe is shown in Figure 32 and it consists of three components. The left end is the recognition unit containing an epoxy-succinyl group, which specifically binds to lysosomes. The middle section is the cell-penetrating peptide (rR)3R2, which assists the fluorescent probe in penetrating the cell membrane. The right end is linked to the fluorescent molecule ATTO 565.

**Figure 33:**
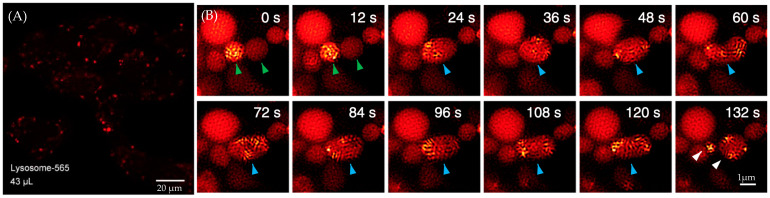
(**A**) Penetration of the ATTO 565 probe into the cell population after 30 min. (**B**) Lysosomal fission–fusion process captured by SIM. Green arrows indicate two lysosomes, while blue arrows mark the fused lysosome. Re-used with permission from [65].

This fluorescent probe exhibits good cell permeability. As shown in Figure 33A, the fluorescent probe effectively enters the cells after a 30 min incubation with live cell cultures. Cytotoxicity assays also indicate that 85% of the cells remain viable after 30 min of incubation. SIM series images demonstrate the process of lysosomal fission–fusion labeled by ATTO 565 (Figure 33B).

#### 3.5.2. ATTO 565 in Fluorescence Lifetime Imaging Microscopy (FLIM)

**Figure 34:**
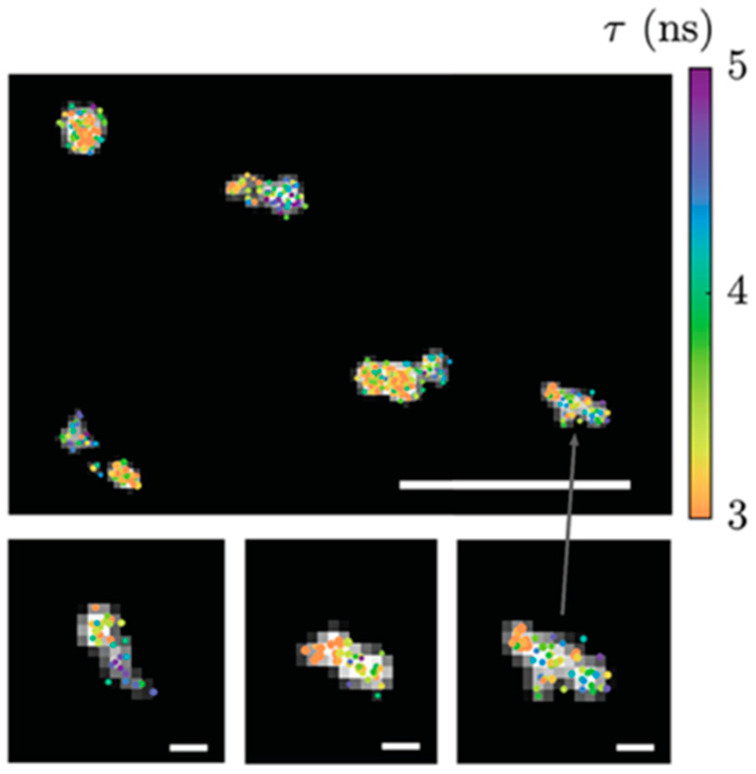
FLIM images of DNA origami samples stained in dual channels, scale bar 1 μm, insets 100 nm. Re-used with permission from [66].

Fluorescence Lifetime Imaging Microscopy (FLIM) measures the time it takes for fluorescent molecules to return from the excited state to the ground state, providing richer molecular information compared to traditional fluorescence microscopy. This is particularly useful for detecting subtle changes in chemical environments. FLIM employs short-pulsed lasers with pulse widths in the picosecond (ps) to nanosecond (ns) range as the excitation source, and uses high-time-resolution photomultiplier tubes to capture fluorescence photons for accurate measurement of the fluorescence signal decay process. ATTO 565 is also suitable for this microscopy technique. Bowman et al. use DNA point accumulation in nanoscale topography (DNA-PAINT) to make super-resolution imaging of DNA origami samples by wide-field FLIM and an ATTO 565 fluorescent probe [66]. Cy3B and Atto 565 labeling on DNA origami structures reveals lifetime differences at binding sites along the structures, as shown in Figure 34.

## 4. Future Prospects

The research presented in this review highlights the primary application of ATTO 565 within the field of biochemistry. ATTO 565 is predominantly employed for the imaging of biological structures across a wide range of scenarios. However, there is a noticeable scarcity of studies addressing the biocompatibility of ATTO 565. Therefore, in the future, it would be beneficial to conduct more extensive investigations into the biocompatibility of ATTO 565. This could encompass experiments to assess the toxicity of ATTO 565 on life cells, examine whether ATTO 565 has the potential to induce gene mutations or genetic toxicity, and evaluate the biodegradability of ATTO 565. The findings from these experiments would contribute to better elucidating the potential impact of the fluorescent dye itself on biological structures during bio-imaging experiments.

**Figure 35:**
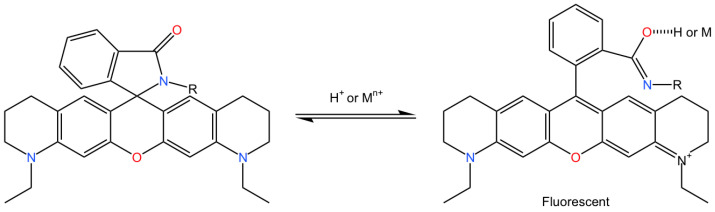
The chemical reaction equation for the hypothetical alteration in ATTO 565’s structure to make it responsive to metal ions. Nitrogen in blue, oxygen in red, ammonium and carbon is black. Reprinted (adapted) with permission from [67].

Apart from its applications in the field of biological labeling, ATTO 565 may have the ability to undergo reactions with metal ions following structural modifications. Some Rhodamine dyes could change between closed form and open form as long as there is a nitrogen atom in proximity to the xanthene ring, as shown in Figure 35 [67]. It is believed that if the structure of ATTO 565 is slightly modified by replacing the oxygen atom of its carboxyl group with a nitrogen atom through organic chemistry methods, ATTO 565 can also exhibit properties of altering its conformation in response to the action of metal ions and emit fluorescence. Limited research has explored this application; thus, the authors believe that ATTO 565 holds promise for the detection of environmental heavy metal ions.

To achieve this concept, the first step is to determine the appropriate organic reaction pathway to modify the ATTO 565 molecule. It should also be determined whether metal ions of different types, but with the same concentration, have varying degrees of impact on the fluorescence intensity of ATTO 565. Subsequently, the fluorescence intensity response curve of ATTO 565 to different concentrations of metal ions should be determined. The fundamental principle of using ATTO 565 to detect the content of environmental metal ions involves mixing the environmental sample with an ATTO 565 solution and subsequently measuring the fluorescence intensity using a fluorescence spectrophotometer. The fluorescence intensity emitted by ATTO 565 is directly proportional to the metal ion content. Finally, the potential interactions between ATTO 565 and various metal catalysts should be determined.

In conclusion, there is a need for further research on the biocompatibility and metal ion detection aspects of ATTO 565. Recent work has shown that ATTO 565 can be used to visualize bone apatite nanofibers by using confocal microscopy [68].

## 5. Conclusions

The chemical and optical characteristics of ATTO 565 unveil the outstanding fluorescent properties of this Rhodamine system. ATTO 565 can be applied in confocal microscopy for the detailed observation of microstructure and chemical bonding on cell membranes. ATTO 565 is very suitable for 2D and 3D single-molecule tracking, FCS, CW STED, and T-Rex STED, pushing the resolution of microscopy to new heights.

The research exemplified in this review demonstrates that ATTO 565 is a highly versatile fluorescent dye. Beyond fulfilling the functions like other fluorescent dyes, ATTO 565 can serve as a flexible scaffold, allowing researchers to modify its structure or combine it with other molecules, endowing ATTO 565 with a multitude of novel properties. In summary, the excellent fluorescence performance of ATTO 565 and its multiple tunable sites make it a fluorescent dye worthy of in-depth investigation and research application.

ATTO 565 has demonstrated extensive adaptability across various microscopy techniques, yet it has predominantly been utilized in studies involving fixed cells. It is widely recognized that observing living samples provides more dynamic and informative results. However, ATTO 565’s application in live samples remains limited. This constraint highlights the need for chemical modifications to enhance its bio-compatibility, thereby significantly expanding its potential applications.

## Figures and Tables

**Table 1:** The optical data of ATTO 565 [14].

λ_abs_ (nm)	564
ε_max_ (M^−1^cm^−1^)	1.2 × 10^5^
λ_fl_ (nm)	590
Φ_fl_ (%)	90
τ_fl_ (ns)	4.0

**Table 2:** The diffusion time of ATTO 565 in different concentrations of AAc [64].

AAc Concentration (%)	Diffusion Time (s)
0.25	4.51 × 10^−5^
2.5	5.27 × 10^−5^
5	5.76 × 10^−5^
8	6.53 × 10^−5^
9	7.12 × 10^−5^
10	7.34 × 10^−5^

## Abbreviations and Symbols

A_2A_R	Adenosine A2A receptor
APEC	A2A-AR agonist
ACF	Autocorrelation function
CW	Continue wave
CCD	Charge-coupled device
CEM-A cells	Acute lymphoblastic leukemia, All cells
CT	Cut tail
CL	Cylindrical lens
Env	Envelope glycoprotein
FCS	Fluorescence correlation spectroscopy
F-actin	Filamentous actin
FWHM	Full-width-half-maximum
GPCRs	G-protein receptors
GFP	Green fluorescent protein
HOS	Hos osteoblast- like cells
HA	hydroxyapatite
His-tag	histidine residues tag
IC50	Half maximal inhibitory concentration
KD	Dissociation constant
lgG	Immunoglobulin G
LAP	Linear assignment problem
LSM	Light sheet microscope
NA	Numerical aperture
OEG	oligo ethylene glycol
PtK2	Potorous tridactylus kidney epithelial cells
PBW	Proton Beam Writing
PCL	Poly-ε-caprolactone (PCL) materials
PBS	Phosphate-buffered saline
PP	Polyproline
PSF	Point spread function
ROI	Region of Interest
STED	Stimulated emission depletion microscopy
SEM	Scanning electron microscope
Sf9	Spodoptera frugiperda
SMT	Single-molecule tracking
S/N	Signal to noise ratio
T-Rex	Triplet-state relaxation
tris-NTA	tris(hydroxymethyl)aminomethane-nitrilotriacetic acid
WGA	Wheat germ agglutinin
WT	Wild type
An^−^	Anion
λ_abs_	Absorption wavelength
ε_max_	Molar extinction coefficient at maximum absorption wavelength
λ_fl_	Fluorescence peak wavelength
Φ	Fluorescence quantum yield
τ	Fluorescence lifetime
S_0_	Ground state
S_1_	First excited singlet state
T_1_	First triplet state
K_isc_	Intersystem crossing rate
K_rad_	Radiative decay rate
K_exc_	Excitation rate
K_ic_	Internal conversion rate constant
K_tun_	Radiative decay rate constant
K_t_	Total decay rate constant
K_s_	Radiative decay rate Constant
K_ex_	Non-radiative decay rate Constant
N_eff_	Average number of targets in ROI
Τ_diff_	Diffusion time of targets in ROI
D	Dark state
G_αβγ_	G protein heterotrimer α, β and γ
Δr	Full-width-half-maximum
β_rev_	Reversal efficiency
